# Disease Stage-Dependent Association Between Nephrotic-Range Proteinuria and Severe Acute Kidney Injury in Patients with Liver Cirrhosis

**DOI:** 10.3390/jcm15103602

**Published:** 2026-05-08

**Authors:** Gi Jeong Park, Seong Gyu Kim, Sang Gyu Kwak

**Affiliations:** 1 Department of Neurosurgery, Daegu Catholic University School of Medicine, Daegu 42472, Republic of Korea; park0904@cu.ac.kr; 2 Division of Nephrology, Department of Internal Medicine, Daegu Catholic University School of Medicine, Daegu 42472, Republic of Korea; ksgnephro@cu.ac.kr; 3 Department of Medical Statistics, Daegu Catholic University School of Medicine, Daegu 42472, Republic of Korea

**Keywords:** acute kidney injury, Child–Pugh score, liver cirrhosis, nephrotic syndrome, proteinuria, retrospective studies, risk assessment

## Abstract

**Background**: Acute kidney injury (AKI) is a common and serious complication in patients with liver cirrhosis and is associated with poor outcomes. However, whether the association between nephrotic-range proteinuria (NRP) and severe AKI varies by liver disease severity remains unclear. **Methods**: This retrospective cohort study included 408 adult patients with cirrhosis stratified by Child–Pugh class (A, B, and C). Severe AKI was defined as Kidney Disease: Improving Global Outcomes stage 2–3. Multivariable logistic regression analyses were performed in the overall cohort and within each class, with the additional evaluation of interaction effects. **Results**: The incidence of severe AKI increased from 18.4% in class A to 38.8% in class C. In the extended multivariable model incorporating hemodynamic and inflammatory variables, nephrotic-range proteinuria was not significantly associated with severe AKI. In stratified analyses, a significant association was observed only in Child–Pugh class A. Additional analyses suggested that this relationship was attenuated after accounting for sepsis and systemic severity. **Conclusions**: Although NRP prevalence was similar across Child–Pugh classes, the association between NRP and severe AKI appeared to vary by disease stage, particularly before adjustment for systemic severity.

## 1. Introduction

Liver cirrhosis represents the end stage of chronic liver disease and is characterized by progressive fibrosis, portal hypertension, and profound systemic circulatory dysfunction. As cirrhosis advances, patients develop a hyperdynamic circulatory state with systemic vasodilation, reduced effective arterial blood volume, and activation of neurohumoral systems, including the renin–angiotensin–aldosterone system and sympathetic nervous system. These pathophysiological alterations impair renal perfusion and increase susceptibility to kidney injury [1,2,3].

Acute kidney injury (AKI) is one of the most frequent and clinically significant complications in patients with cirrhosis, with reported incidence ranging from 20% to over 50% in hospitalized patients depending on clinical settings and diagnostic criteria [4,5]. The development of AKI is strongly associated with adverse outcomes, including prolonged hospitalization, increased healthcare utilization, and substantially elevated short- and long-term mortality [6,7,8]. Even mild forms of AKI have been shown to negatively impact survival, underscoring the importance of early risk identification and prevention strategies [9,10].

The pathogenesis of AKI in cirrhosis is multifactorial and involves a complex interplay between systemic hemodynamic disturbances and precipitating factors such as infections, gastrointestinal bleeding, hypovolemia, and exposure to nephrotoxic agents [11,12,13]. In this context, the severity of liver dysfunction plays a central role in determining renal vulnerability. The Child–Pugh classification is widely used in clinical practice to assess liver disease severity and predict prognosis. Progressive worsening from Child–Pugh class A to C is associated with increasing circulatory dysfunction, systemic inflammation, and impaired renal autoregulation, all of which contribute to a higher risk of AKI and poorer clinical outcomes [9,14].

Despite extensive research, most previous studies have evaluated AKI in cirrhotic patients using pooled cohorts and have treated liver disease severity primarily as a covariate in regression models [5,11,12]. Such approaches implicitly assume that the effects of risk factors are homogeneous across disease stages. However, this assumption may not be valid, as the relative contribution of individual risk factors is likely to vary according to the degree of hepatic dysfunction. Consequently, conventional analytical frameworks may obscure important heterogeneity in the underlying risk structure of AKI.

Nephrotic-range proteinuria (NRP), defined as a urine protein-to-creatinine ratio ≥3.5 g/g, is a marker of significant glomerular injury and systemic disease burden. It is associated with hypoalbuminemia, reduced plasma oncotic pressure, intravascular volume depletion, and endothelial dysfunction, all of which may exacerbate renal hypoperfusion and predispose patients to AKI. In addition, proteinuria reflects underlying renal structural damage and inflammatory activation, further increasing susceptibility to AKI. Previous studies have suggested that proteinuria and renal biomarkers are associated with AKI development in cirrhotic patients [15]; however, these studies have not specifically evaluated whether the association of proteinuria with AKI differs according to liver disease severity.

Recent evidence suggests that hepatorenal syndrome is not merely a hemodynamic disorder but a complex condition involving both circulatory dysfunction and systemic inflammation. Portal hypertension-induced splanchnic vasodilation leads to reduced effective arterial blood volume and the activation of vasoconstrictor systems, ultimately resulting in renal hypoperfusion. In addition, systemic inflammation and endothelial dysfunction further contribute to renal microcirculatory impairment and kidney injury [16,17,18].

Given the dynamic changes in hemodynamic status and systemic inflammation across stages of cirrhosis, it is plausible that the clinical significance of proteinuria varies depending on disease severity. A stage-specific assessment may therefore provide more precise risk stratification and improve the identification of high-risk patients.

Therefore, the present study aimed to (1) determine the incidence and risk factors of severe AKI within each Child–Pugh class, and (2) evaluate whether the association between nephrotic-range proteinuria and severe AKI differs according to liver disease severity. Through this framework, we sought to characterize potential heterogeneity in the relationship between proteinuria and AKI according to liver disease severity and to provide clinically relevant insights for stage-specific risk assessment.

## 2. Materials and Methods

### 2.1. Study Design and Population

This retrospective observational cohort study was conducted at a single tertiary referral center, Daegu Catholic University Medical Center in Korea. We included adult patients aged 18 years or older who were hospitalized with a diagnosis of liver cirrhosis between 1 January 2016 and 31 December 2025. Liver cirrhosis was identified using International Classification of Diseases, 10th Revision (ICD-10) diagnostic codes in combination with clinical or imaging confirmation documented in the electronic medical records. To ensure consistency, patients were included only when the diagnosis of cirrhosis was supported by either imaging findings or specialist documentation in addition to diagnostic coding. For patients with multiple hospital admissions during the study period, only the first hospitalization (index admission) was included in the analysis to avoid duplication and potential within-patient correlation. Patients were excluded if they were receiving maintenance renal replacement therapy, including hemodialysis or peritoneal dialysis, at baseline, or if baseline serum creatinine values required for the assessment of AKI were unavailable. In addition, patients with missing data required for the definition of NRP or AKI staging were excluded, and a complete-case analysis was performed. Therefore, only patients with complete data on proteinuria and AKI were included in the final analysis. All data were extracted from the electronic medical records and the institutional clinical data warehouse system. Patient identifiers were anonymized prior to analysis to ensure confidentiality.

This study was approved by the Institutional Review Board of Daegu Catholic University Medical Center (approval number: DCUMC-2026-03-010). The requirement for informed consent was waived due to the retrospective nature of the study. All procedures were conducted in accordance with the principles of the Declaration of Helsinki.

### 2.2. Definitions and Measurements

The severity of liver disease was assessed using the Child–Pugh classification, which incorporates serum bilirubin, albumin, prothrombin time expressed as the international normalized ratio (INR), and the presence of ascites and hepatic encephalopathy. Patients were categorized into Child–Pugh class A, B, or C based on these criteria. Baseline renal function was defined using pre-admission serum creatinine values. Specifically, the baseline creatinine level was defined as the lowest serum creatinine measured within seven days prior to hospital admission. If no measurement was available within this period, the lowest value obtained within the preceding three months in a clinically stable condition (i.e., in the absence of acute illness, infection, or hospitalization) was used as the baseline value. To evaluate the robustness of this baseline definition, sensitivity analyses were performed using alternative definitions, including the median pre-admission creatinine and the last available stable creatinine prior to admission. Severe AKI was reclassified according to each definition, and multivariable logistic regression analyses were repeated using the same covariates as in the primary analysis. AKI was defined according to the Kidney Disease: Improving Global Outcomes (KDIGO) serum creatinine criteria [9,19] and was assessed throughout the entire hospitalization period using all available serum creatinine measurements. AKI staging was determined based on the relative increase in serum creatinine from baseline, with stage 1 defined as a 1.5–1.9-fold increase or an absolute increase of at least 0.3 mg/dL; stage 2 as a 2.0–2.9-fold increase; and stage 3 as a threefold or greater increase, a serum creatinine level of at least 4.0 mg/dL, or the initiation of renal replacement therapy. Severe AKI was defined as KDIGO stage 2 or 3. Nephrotic-range proteinuria (NRP) was defined as a urine protein-to-creatinine ratio (UPCR) of 3.5 g/g or greater.

Demographic and clinical variables included age and sex. Comorbidities, including diabetes mellitus, hypertension, and chronic kidney disease (CKD), were identified based on diagnostic codes and clinical records. CKD was defined as a documented history of CKD or an estimated glomerular filtration rate <60 mL/min/1.73 m^2^ prior to admission. Sepsis at admission was defined as documented infection with evidence of organ dysfunction, based on the Sepsis-3 criteria, as documented in clinical records. Laboratory variables included serum albumin, total bilirubin, INR, and baseline serum creatinine. Additional variables reflecting hemodynamic and inflammatory status were also collected, including mean arterial pressure, vasopressor use, lactate levels, and C-reactive protein.

### 2.3. Outcomes

The primary outcome of this study was the development of severe AKI, defined as KDIGO stage 2 or 3, occurring at any time during the index hospitalization. Secondary outcomes included the occurrence of any AKI, defined according to KDIGO criteria, and the distribution of AKI stages (stage 0–3) during hospitalization.

### 2.4. Statistical Analysis

Continuous variables were presented as the mean with standard deviation or median with interquartile range, depending on their distribution, while categorical variables were expressed as frequencies and percentages. Comparisons among Child–Pugh classes were performed using analysis of variance or the Kruskal–Wallis test for continuous variables and the chi-square test or Fisher’s exact test for categorical variables. To identify risk factors for severe AKI, logistic regression analyses were performed. Univariable analyses were initially conducted to explore associations between individual variables and the outcome. Multivariable logistic regression models were then constructed by including clinically relevant covariates selected a priori based on clinical importance. The primary multivariable model included age, sex, diabetes mellitus, CKD, sepsis, serum albumin, total bilirubin, INR, baseline serum creatinine, NRP, and Child–Pugh class. An extended multivariable model was then constructed by incorporating hemodynamic and inflammatory variables, including mean arterial pressure, vasopressor use, lactate levels, and C-reactive protein. To evaluate stage-specific associations, separate multivariable logistic regression analyses were performed within each Child–Pugh class (A, B, and C). Adjusted odds ratios (ORs) with 95% confidence intervals (CIs) were reported. To further assess whether the association between NRP and severe AKI differed according to liver disease severity, an interaction term between NRP and Child–Pugh class was incorporated into a multivariable logistic regression model using the overall cohort. Multicollinearity was assessed using variance inflation factors, and model fit was evaluated using the Hosmer–Lemeshow goodness-of-fit test and C-statistics. A complete-case analysis was performed due to the exclusion of patients with missing values in key variables. To further evaluate the potential confounding effect of sepsis, additional analyses were performed. These included subgroup analyses stratified by the presence of sepsis, sensitivity analyses excluding patients with sepsis, and interaction analysis between nephrotic-range proteinuria and sepsis. Additional analyses were performed to evaluate clinically relevant outcomes, including the need for renal replacement therapy and length of hospital stay, using multivariable regression models. Given the number of outcome events relative to the number of covariates, we interpreted the model estimates with caution, particularly for variables with wide confidence intervals. A two-sided *p*-value of less than 0.05 was considered statistically significant. All statistical analyses were performed using R software (version 4.5.1; R Foundation for Statistical Computing, Vienna, Austria).

## 3. Results

### 3.1. Baseline Characteristics

**Figure 1:**
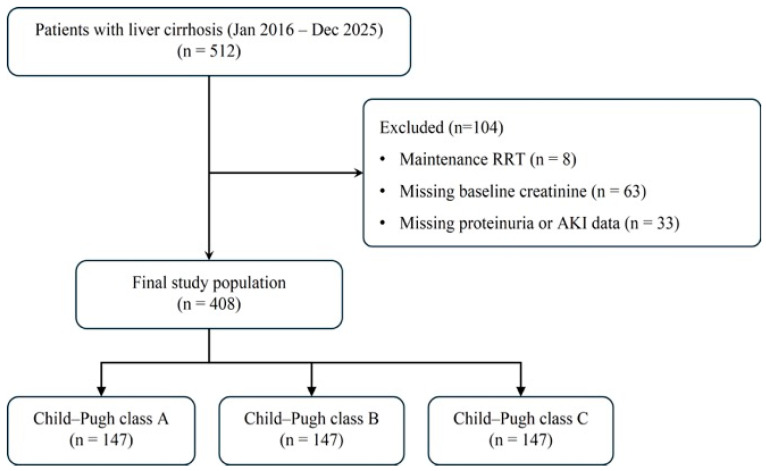
Flow diagram of patient selection. Patients with liver cirrhosis were identified and screened according to predefined inclusion and exclusion criteria. The final study population consisted of 408 patients, who were stratified according to Child–Pugh classification. RRT, renal replacement therapy.

A total of 408 patients with liver cirrhosis were included in the final analysis and categorized according to Child–Pugh class (Figure 1). The distribution of patients was as follows: 147 in class A, 176 in class B, and 85 in class C. The baseline characteristics of the study population are summarized in Table 1. Demographic characteristics and comorbidities, including age, sex, diabetes mellitus, hypertension, CKD, and sepsis at admission, were similar across the three groups. Hemodynamic and inflammatory variables also differed across Child–Pugh classes, with lower mean arterial pressure and higher vasopressor use, lactate levels, and C-reactive protein observed in more advanced disease (Table 1). In contrast, markers of liver disease severity differed significantly according to Child–Pugh class. Serum albumin levels progressively decreased, whereas total bilirubin and INR levels increased from class A to C (all *p* < 0.001). Baseline renal function, as assessed by serum creatinine, did not differ significantly among the groups. Likewise, urine protein-to-creatinine ratio and the prevalence of NRP were comparable across Child–Pugh classes. Therefore, any stage-dependent findings regarding NRP should be interpreted as differences in its association with severe AKI rather than differences in the distribution of proteinuria across Child–Pugh classes.

### 3.2. Incidence and Severity of AKI

**Figure 2:**
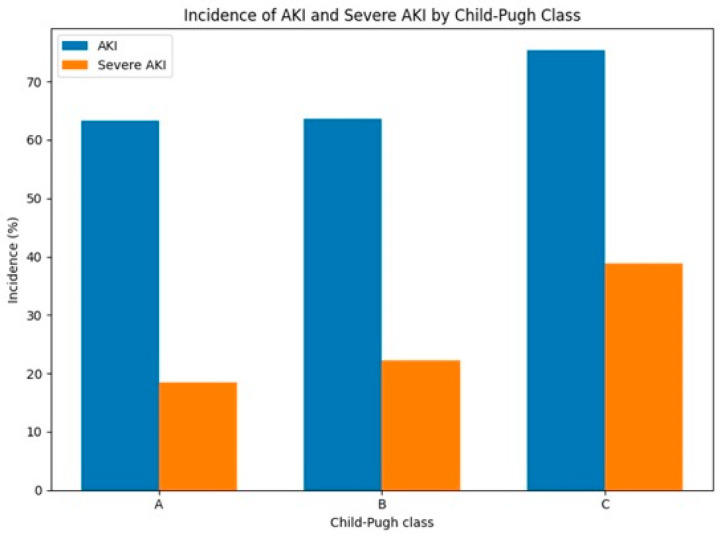
Incidence of acute kidney injury (AKI) and severe AKI according to Child–Pugh class. Severe AKI was defined as Kidney Disease: Improving Global Outcomes (KDIGO) stage 2–3.

The incidence of AKI was 63.3%, 63.6%, and 75.3% in Child–Pugh classes A, B, and C, respectively (Figure 2 and Table 2). The incidence was similar between classes A and B and appeared higher in class C. The incidence of severe AKI increased across Child–Pugh classes, from 18.4% in class A to 22.2% in class B and 38.8% in class C. In addition, the distribution of AKI stages shifted toward greater severity with worsening liver function, with a higher proportion of stage 2 and stage 3 AKI observed in patients with Child–Pugh class C. UPCR levels and the prevalence of nephrotic-range proteinuria did not differ significantly across AKI stages (Supplementary Table S5), and no clear graded pattern was observed. These findings suggest that proteinuria was not associated with increasing AKI severity. Because hemodynamic instability and systemic inflammation may contribute to the higher incidence of severe AKI in advanced cirrhosis, related variables including mean arterial pressure, vasopressor use, lactate levels, and C-reactive protein were summarized in Table 1 and further incorporated into the extended multivariable model.

### 3.3. Risk Factors for Severe AKI in the Overall Cohort

In univariable analyses, several clinical and laboratory variables were associated with severe AKI, including CKD, ICU admission, ascites, and Child–Pugh class C (Table 3). In the extended multivariable logistic regression analysis incorporating hemodynamic and inflammatory variables, including mean arterial pressure, vasopressor use, lactate levels, and C-reactive protein, nephrotic-range proteinuria was not significantly associated with severe AKI (adjusted odds ratio [OR] 0.90, 95% CI 0.45–1.81, *p* = 0.772). Although some estimates showed relatively wide confidence intervals, the direction of the associations was consistent across models. Among the variables included in the model, total bilirubin and Child–Pugh class C remained significantly associated with severe AKI. Sepsis was not independently associated with severe AKI after adjustment for hemodynamic and inflammatory variables. Sensitivity analyses using alternative baseline creatinine definitions yielded consistent results (Supplementary Table S4). The incidence of severe AKI remained similar across definitions, and the association between nephrotic-range proteinuria and severe AKI showed nearly identical effect estimates with overlapping confidence intervals. Notably, the crude incidence of severe AKI was similar between patients with and without nephrotic-range proteinuria (25.5% vs. 24.1%). In additional analyses evaluating clinically relevant outcomes, nephrotic-range proteinuria was not significantly associated with the need for renal replacement therapy or length of hospital stay after adjustment for hemodynamic and inflammatory variables (Supplementary Tables S2 and S3). Lactate level showed a positive association with the need for RRT, although this analysis should be interpreted cautiously given the limited number of RRT events and wide confidence intervals.

### 3.4. Stratified Analysis According to Child–Pugh Class

**Figure 3:**
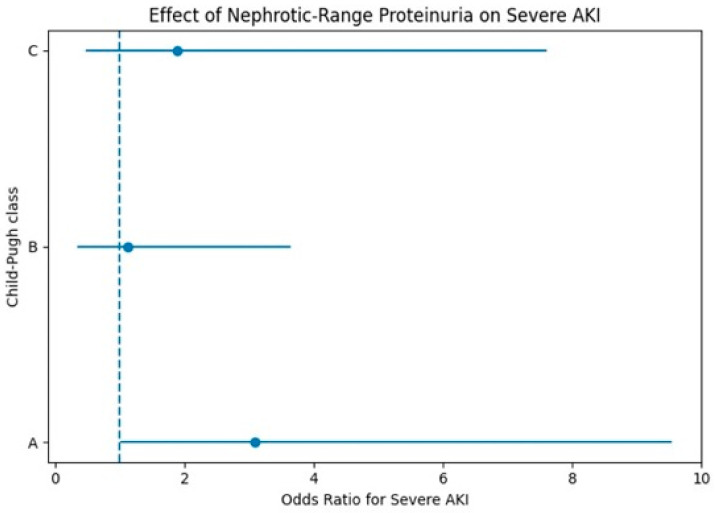
Adjusted odds ratios for the association between nephrotic-range proteinuria and severe AKI in separate multivariable logistic regression models stratified by Child–Pugh class. Points represent odds ratios, and horizontal lines indicate 95% confidence intervals. The dashed vertical line indicates an odds ratio of 1.

To evaluate associations according to liver disease severity, multivariable logistic regression analyses were performed separately within each Child–Pugh class (Table 4, Figure 3). NRP was associated with an increased risk of severe AKI in patients with Child–Pugh class A (adjusted OR 3.09, 95% CI 1.00–9.54, *p* = 0.049), although this finding should be interpreted cautiously given the wide confidence interval and limited sample size. In contrast, no statistically significant association was observed between NRP and severe AKI in patients with Child–Pugh class B or C.

Sepsis was not significantly associated with severe AKI in class A, whereas it was independently associated with severe AKI in classes B and C. Total bilirubin showed a positive association with severe AKI in class A, whereas an inverse association was observed in class C. However, this finding should be interpreted with caution, as it may reflect statistical instability or residual confounding rather than a true protective effect.

### 3.5. Interaction Analysis

**Figure 4:**
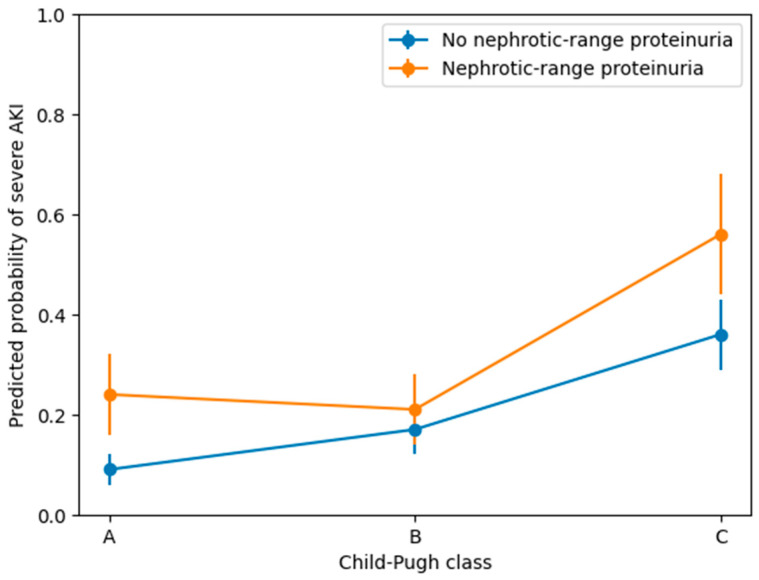
Predicted probabilities with 95% confidence intervals were derived from the extended multivariable logistic regression model. Overlapping confidence intervals indicate no statistically significant differences between groups.

To further assess whether the association between NRP and severe AKI differed according to liver disease severity, an interaction analysis was performed using the overall cohort (Table 5). In the overall model, NRP was not significantly associated with severe AKI in the multivariable analysis, and the effect estimate was small with a wide confidence interval, indicating uncertainty. However, the interaction terms between NRP and Child–Pugh class were not statistically significant. These findings suggest that although the magnitude of the association appeared to vary across Child–Pugh classes, there was no statistically significant interaction. The predicted probability of severe AKI according to Child–Pugh class and NRP is presented in Figure 4. To further assess the potential confounding effect of sepsis, additional analyses were performed (Supplementary Table S1). In subgroup analyses, nephrotic-range proteinuria was not significantly associated with severe AKI in patients without sepsis (OR 0.92, 95% CI 0.43–1.97, *p* = 0.823) or in those with sepsis (OR 0.77, 95% CI 0.13–4.52, *p* = 0.768). Similarly, in sensitivity analyses excluding patients with sepsis, NRP was not associated with severe AKI (OR 0.92, 95% CI 0.43–1.97, *p* = 0.823). In the interaction analysis, the interaction term between NRP and sepsis was not statistically significant (*p* = 0.780).

## 4. Discussion

In this retrospective cohort study of patients with liver cirrhosis, we evaluated the relationship between NRP and severe AKI across different stages of liver disease. Our findings suggest that severe AKI was more frequent in advanced liver disease and that the association between NRP and severe AKI appeared to differ according to Child–Pugh class. However, this association was attenuated after accounting for sepsis and systemic severity, suggesting that NRP should be interpreted as a potential risk marker rather than an independent causal factor.

The association between worsening liver function and increased risk of AKI observed in our study is consistent with previous literature. Progressive cirrhosis is characterized by severe circulatory dysfunction, including splanchnic vasodilation, reduced effective arterial blood volume, and activation of vasoconstrictor systems, which together impair renal perfusion and predispose patients to AKI [1,3,9]. In line with prior studies, we observed a higher incidence of severe AKI in patients with Child–Pugh class C, reinforcing the concept that advanced liver disease is a major determinant of renal vulnerability [4,5,11].

The pathophysiology of hepatorenal syndrome has evolved from a purely hemodynamic concept to a multifactorial process involving both circulatory dysfunction and systemic inflammation. Splanchnic arterial vasodilation and reduced effective arterial blood volume lead to compensatory activation of vasoconstrictor systems, resulting in marked renal vasoconstriction and hypoperfusion. More recent evidence highlights the role of systemic inflammation, bacterial translocation, and endothelial dysfunction in exacerbating renal injury. These findings suggest that kidney dysfunction in cirrhosis reflects a complex interplay between hemodynamic and inflammatory mechanisms rather than a single causal pathway [16,18,20].

Nephrotic-range proteinuria has been increasingly recognized as a marker of renal structural damage and systemic disease burden. Previous studies have suggested that proteinuria and renal biomarkers are associated with the development of AKI in patients with cirrhosis [15]. In the present study, NRP was not significantly associated with severe AKI in the extended multivariable analysis.

A key observation of this study is that the association between NRP and severe AKI varied across stages of liver disease. Importantly, UPCR levels and the prevalence of NRP were comparable across Child–Pugh classes at the baseline.

Although a crude association between NRP and severe AKI was observed, this relationship was attenuated after adjustment for sepsis and hemodynamic and inflammatory variables. This suggests that the observed association may be driven, at least in part, by systemic illness severity rather than reflecting an independent causal effect. The association between NRP and severe AKI appeared to vary according to disease stage, although no statistically significant interaction was observed. UPCR levels and the prevalence of nephrotic-range proteinuria did not differ across AKI stages, indicating that proteinuria was not associated with AKI severity.

Several mechanisms may explain this finding. In early-stage cirrhosis, renal function is relatively preserved and systemic hemodynamic disturbances are less pronounced. In this setting, markers of intrinsic renal vulnerability, such as proteinuria, may play a more prominent role in determining susceptibility to AKI. NRP may reflect underlying glomerular injury, endothelial dysfunction, and subclinical renal damage, thereby increasing susceptibility to acute insults. In contrast, in advanced cirrhosis, severe circulatory dysfunction and systemic inflammation are dominant drivers of renal injury. Under these conditions, the relative contribution of proteinuria may be attenuated by factors such as profound vasodilation, reduced renal perfusion, and infection-related hemodynamic instability. The inverse association observed for total bilirubin in advanced cirrhosis should be interpreted with caution, as it may reflect statistical instability or residual confounding rather than a true protective effect.

Importantly, although stratified analyses suggested differences across Child–Pugh classes, formal interaction analysis did not demonstrate statistically significant effect modification. Therefore, these findings should be interpreted as exploratory. This may reflect limited statistical power or insufficient sample size within subgroups. Differences in effect estimates between models may be attributable to differences in model specification, particularly the inclusion of interaction terms. Overall, these results are best viewed as suggestive of heterogeneity rather than definitive evidence of effect modification.

The findings of this study have important clinical implications. First, the prognostic value of proteinuria in cirrhotic patients may not be uniform across disease stages. In particular, the presence of NRP in patients with early-stage cirrhosis may help identify individuals at increased risk of severe AKI; however, this finding should be interpreted with caution.

Renal dysfunction is increasingly recognized across a spectrum of chronic liver diseases beyond cirrhosis. Recent studies in patients with nonalcoholic fatty liver disease have also demonstrated a substantial burden of impaired renal function, suggesting that kidney involvement may reflect broader systemic and metabolic disturbances associated with liver disease [21].

Additional analyses of clinically relevant outcomes demonstrated that nephrotic-range proteinuria was not associated with the need for renal replacement therapy or length of hospital stay (Supplementary Tables S2 and S3). These findings further support the interpretation that proteinuria reflects overall systemic disease severity rather than serving as an independent prognostic factor. In contrast, lactate, a marker of systemic hypoperfusion, was associated with the need for renal replacement therapy (Supplementary Table S2), highlighting the importance of hemodynamic and metabolic factors in severe renal outcomes.

This study has several limitations. First, it was conducted at a single center, which may limit the generalizability of the findings. Second, the retrospective design introduces the possibility of residual confounding despite multivariable adjustment. In particular, the higher incidence of severe AKI observed in Child–Pugh class C may partly reflect greater hemodynamic instability, systemic inflammation, or illness severity rather than liver disease severity alone. Although variables reflecting hemodynamic and inflammatory status, including mean arterial pressure, vasopressor use, lactate levels, and C-reactive protein, were incorporated into the extended multivariable model, residual confounding by unmeasured or incompletely captured factors such as fluid balance, diuretic exposure, albumin infusion, and dynamic changes in sodium or circulatory status cannot be excluded. Unmeasured factors such as hemodynamic status, medication use, and infection severity may have influenced the results. Third, the use of complete-case analysis may have introduced selection bias if missingness was not random. In particular, patients with greater clinical instability, fluid overload, or incomplete monitoring may have been less likely to have available data on proteinuria or AKI and could be at higher risk of adverse renal outcomes. Therefore, the study population may not fully represent the most critically ill patients, and the findings should be interpreted with caution. Fourth, the sample size, particularly within subgroup analyses, may have limited statistical power and resulted in imprecise estimates. In particular, the number of severe AKI events relative to the number of covariates included in the multivariable models may have led to statistical instability and wide confidence intervals for some estimates. Therefore, the observed associations, especially those with borderline statistical significance, should be interpreted with caution. This limitation is particularly relevant for subgroup analyses, where inconsistent or paradoxical associations may arise due to reduced statistical power and model instability. Fifth, proteinuria was assessed based on a single UPCR measurement, which may have led to misclassification. In patients with cirrhosis, proteinuria may fluctuate due to changes in volume status, infections, diuretic use, and hemodynamic conditions. Therefore, the distinction between persistent glomerular proteinuria and transient or functional proteinuria could not be fully established. Accordingly, the observed association between nephrotic-range proteinuria and severe AKI should be interpreted with caution, as proteinuria in this setting may reflect dynamic clinical conditions rather than a stable underlying renal phenotype. The use of the lowest creatinine value to define baseline kidney function may have underestimated baseline renal function and potentially overestimated AKI incidence, particularly in cirrhotic patients with dynamic volume status. However, sensitivity analyses using alternative baseline definitions demonstrated highly consistent results, suggesting that this potential bias had minimal impact on our findings. Finally, external validation was not performed, and the generalizability of the findings requires confirmation in independent cohorts.

Despite these limitations, this study has notable strengths. By applying a stratified analytical framework based on Child–Pugh classification, we were able to explore potential heterogeneity in the association between NRP and AKI. In addition, the combined use of stratified and interaction analyses provides a comprehensive assessment of this relationship and supports the clinical relevance of our findings. Additionally, given the observational nature of the study, causal relationships cannot be established, and the findings should be interpreted as associations rather than evidence of causality.

## 5. Conclusions

In conclusion, although UPCR levels and NRP prevalence were comparable across Child–Pugh classes, the association between NRP and severe AKI was observed primarily in early-stage cirrhosis. However, this association was attenuated after accounting for sepsis and systemic illness severity, suggesting that NRP should be interpreted as a potential risk marker rather than a stage-specific causal factor. These findings suggest potential heterogeneity in the association between proteinuria and AKI according to liver disease severity. Proteinuria may help identify patients at increased risk of AKI in early-stage cirrhosis; however, this observation should be interpreted with caution and requires confirmation in future studies.

## Figures and Tables

**Table 1:** Baseline characteristics of patients according to Child–Pugh class.

Variable	Child–Pugh A (n = 147)	Child–Pugh B (n = 176)	Child–Pugh C (n = 85)	*p* Value
Age, years	64.0 (57.0–71.0)	65.0 (58.0–72.0)	65.0 (59.0–73.0)	0.412
Male sex, n (%)	103 (70.1)	122 (69.3)	53 (62.4)	0.523
DM, n (%)	62 (42.2)	74 (42.0)	36 (42.4)	0.997
HTN, n (%)	63 (42.9)	73 (41.5)	37 (43.5)	0.955
CKD, n (%)	21 (14.3)	30 (17.0)	15 (17.6)	0.693
Sepsis, n (%)	34 (23.1)	44 (25.0)	25 (29.4)	0.486
ICU admission, n (%)	32 (21.8)	45 (25.6)	29 (34.1)	0.081
Albumin, g/dL	3.25 (2.90–3.60)	2.90 (2.50–3.20)	2.50 (2.20–2.90)	<0.001
TB, mg/dL	1.90 (1.30–2.60)	2.70 (1.80–4.10)	4.90 (2.90–8.20)	<0.001
INR	1.20 (1.10–1.40)	1.40 (1.20–1.60)	1.70 (1.40–2.20)	<0.001
Baseline Cr, mg/dL	0.92 (0.75–1.10)	0.98 (0.80–1.20)	1.05 (0.85–1.30)	0.061
UPCR, g/g	1.40 (0.90–2.20)	1.60 (1.00–2.50)	1.80 (1.10–2.90)	0.072
NRP, n (%)	16 (10.9)	21 (11.9)	12 (14.1)	0.721
MAP, mmHg	85.0 (78.0–92.0)	82.0 (75.0–90.0)	78.0 (70.0–86.0)	<0.001
Vasopressor use, n (%)	18 (12.2)	28 (15.9)	22 (25.9)	0.018
Lactate, mmol/L	1.8 (1.2–2.5)	2.1 (1.4–3.0)	2.8 (1.8–4.2)	<0.001
CRP, mg/dL	2.5 (0.8–6.0)	3.2 (1.2–7.8)	4.5 (1.5–10.5)	0.032

Values are presented as median (interquartile range) or number (percentage). Comparisons among groups were performed using the Kruskal–Wallis test for continuous variables and the chi-square test or Fisher’s exact test for categorical variables. CKD, chronic kidney disease; Cr, creatinine; CRP, C-reactive protein; DM, diabetes mellitus; HTN, hypertension; ICU, intensive care unit; INR, international normalized ratio; MAP, mean arterial pressure; NRP, nephrotic-range proteinuria; TB, total bilirubin; UPCR, urine protein-to-creatinine ratio.

**Table 2:** Incidence and severity of acute kidney injury according to Child–Pugh class.

Variable	Child–Pugh A (n = 147)	Child–Pugh B (n = 176)	Child–Pugh C (n = 85)	*p*-Value
AKI	93 (63.3)	112 (63.6)	64 (75.3)	0.123
Severe AKI	27 (18.4)	39 (22.2)	33 (38.8)	0.002
AKI stage distribution				0.008
Stage 0	54 (36.7)	64 (36.4)	21 (24.7)	
Stage 1	66 (44.9)	73 (41.5)	31 (36.5)	
Stage 2	16 (10.9)	30 (17.0)	19 (22.4)	
Stage 3	11 (7.5)	9 (5.1)	14 (16.5)	

Values are presented as number (percentage). Comparisons among groups were performed using the chi-square test. AKI was defined according to the KDIGO serum creatinine criteria. Severe AKI was defined as KDIGO stage 2–3.

**Table 3:** Univariable and extended multivariable logistic regression analysis for severe acute kidney injury.

Variable	Univariable Analysis		Multivariate Analysis	
	OR (95% CI)	*p* Value	OR (95% CI)	*p* Value
Age (year)	1.00 (0.98–1.02)	0.997	1.00 (0.98–1.02)	0.787
Male	0.57 (0.36–0.92)	0.020	0.60 (0.37–0.98)	0.042
DM	1.13 (0.72–1.79)	0.596	1.04 (0.64–1.69)	0.868
HTN	0.76 (0.48–1.21)	0.245	–	–
CKD	0.90 (0.48–1.69)	0.750	0.77 (0.39–1.51)	0.450
Sepsis	0.75 (0.43–1.28)	0.290	0.78 (0.31–1.93)	0.586
ICU admission	1.72 (1.05–2.81)	0.030	–	–
Total bilirubin (mg/dL)	1.01 (0.91–1.13)	0.821	0.84 (0.72–0.98)	0.022
Albumin (g/dL)	0.81 (0.58–1.14)	0.228	1.04 (0.71–1.51)	0.852
INR	1.36 (0.82–2.28)	0.237	0.95 (0.51–1.78)	0.883
Ascites	1.73 (1.09–2.73)	0.019	–	–
Variceal bleeding	1.38 (0.81–2.38)	0.238	–	–
Baseline creatinine (mg/dL)	1.22 (0.55–2.72)	0.621	0.96 (0.41–2.26)	0.929
UPCR (g/g)	1.04 (0.89–1.22)	0.616	–	–
NRP	1.08 (0.56–2.07)	0.825	0.90 (0.45–1.81)	0.772
MAP (mmHg)	1.00 (0.98–1.02)	0.981	1.02 (0.99–1.05)	0.251
Vasopressor use	1.39 (0.75–2.59)	0.293	1.20 (0.54–2.65)	0.652
Lactate (mmol/L)	1.25 (0.92–1.70)	0.152	0.96 (0.59–1.56)	0.860
C-reactive protein (mg/dL)	0.97 (0.90–1.04)	0.350	0.97 (0.89–1.06)	0.496
Child–Pugh class B vs. A	1.27 (0.73–2.19)	0.401	1.74 (0.90–3.35)	0.098
Child–Pugh class C vs. A	2.82 (1.54–5.16)	<0.001	6.83 (2.48–18.82)	<0.001

Odds ratios (ORs) with 95% confidence intervals (CIs) were estimated using logistic regression. The extended multivariable analysis included age, sex, diabetes mellitus, chronic kidney disease, sepsis, albumin, total bilirubin, INR, baseline creatinine, nephrotic-range proteinuria, Child–Pugh class, as well as hemodynamic and inflammatory variables including mean arterial pressure, vasopressor use, lactate levels, and C-reactive protein. CI, confidence interval; CKD, chronic kidney disease; Cr, creatinine; DM, diabetes mellitus; HTN, hypertension; ICU, intensive care unit; INR, international normalized ratio; MAP, mean arterial pressure; NRP, nephrotic-range proteinuria; OR, odds ratio; UPCR, urine protein-to-creatinine ratio.

**Table 4:** Stratified multivariable logistic regression analysis for severe acute kidney injury.

Variable	Child–Pugh A		Child–Pugh B		Child–Pugh C	
	OR (95% CI)	*p* Value	OR (95% CI)	*p* Value	OR (95% CI)	*p* Value
Age (year)	1.01 (0.97–1.06)	0.606	0.97 (0.93–1.01)	0.097	1.00 (0.94–1.05)	0.866
Male	1.07 (0.41–2.85)	0.884	0.58 (0.25–1.33)	0.197	1.29 (0.39–4.29)	0.683
DM	0.37 (0.12–1.13)	0.080	0.75 (0.33–1.72)	0.504	1.17 (0.39–3.49)	0.779
CKD	2.36 (0.49–11.39)	0.285	1.18 (0.31–4.45)	0.811	0.74 (0.11–5.19)	0.762
Sepsis	0.98 (0.35–2.70)	0.963	2.39 (1.09–5.25)	0.031	3.03 (1.04–8.86)	0.042
Albumin (g/dL)	0.86 (0.26–2.80)	0.796	1.61 (0.51–5.03)	0.417	1.73 (0.39–7.70)	0.473
TB (mg/dL)	1.58 (1.01–2.47)	0.043	1.11 (0.81–1.53)	0.517	0.69 (0.48–0.97)	0.035
INR	0.21 (0.01–3.24)	0.263	1.10 (0.11–10.64)	0.933	3.51 (0.19–64.87)	0.399
Baseline Cr (mg/dL)	0.75 (0.09–6.48)	0.797	5.24 (0.72–37.96)	0.101	9.88 (0.74–131.98)	0.083
NRP	3.09 (1.00–9.54)	0.049	1.13 (0.35–3.65)	0.840	1.89 (0.47–7.61)	0.370

Adjusted odds ratios (ORs) with 95% confidence intervals (CIs) were estimated using separate multivariable logistic regression models within each Child–Pugh class. Each model included age, sex, DM, CKD, sepsis, albumin, TB, INR, baseline Cr, and NRP. CI, confidence interval; CKD, chronic kidney disease; Cr, creatinine; DM, diabetes mellitus; INR, international normalized ratio; NRP, nephrotic-range proteinuria; OR, odds ratio; TB, total bilirubin.

**Table 5:** Interaction analysis of nephrotic-range proteinuria and Child–Pugh class for severe acute kidney injury.

Variable	Adjusted OR (95% CI)	*p* Value
NRP	0.60 (0.12–2.92)	0.528
Child–Pugh class B vs. A	1.57 (0.78–3.15)	0.203
Child–Pugh class C vs. A	6.62 (2.32–18.90)	<0.001
NRP × Child–Pugh B	2.17 (0.32–14.54)	0.426
NRP × Child–Pugh C	1.28 (0.18–8.95)	0.807

Adjusted odds ratios (ORs) with 95% confidence intervals (CIs) were estimated using a multivariable logistic regression model including interaction terms between nephrotic-range proteinuria and Child–Pugh class. The model was adjusted for age, sex, diabetes mellitus, chronic kidney disease, albumin, total bilirubin, INR, baseline creatinine, and hemodynamic and inflammatory variables including mean arterial pressure, vasopressor use, lactate levels, and C-reactive protein. CI, confidence interval; NRP, nephrotic-range proteinuria; OR, odds ratio.

## Data Availability

The data presented in this study are available on request from the corresponding author under ethical and legal restrictions related to patient confidentiality.

## Supplementary Materials

The following supporting information can be downloaded at: https://www.mdpi.com/article/10.3390/jcm15103602/s1. Supplementary Table S1. Sensitivity and subgroup analyses for the association between nephrotic-range proteinuria and severe acute kidney injury according to sepsis status. Supplementary Table S2. Multivariable logistic regression analysis for the need for renal replacement therapy incorporating hemodynamic and inflammatory variables. Supplementary Table S3. Multivariable linear regression analysis for length of hospital stay incorporating hemodynamic and inflammatory variables. Supplementary Table S4. Sensitivity analyses using alternative definitions of baseline creatinine. Supplementary Table S5. UPCR levels and prevalence of nephrotic-range proteinuria according to AKI stage.

## Abbreviations

The following abbreviations are used in this manuscript:

AKI	Acute kidney injury
CI	Confidence interval
CKD	Chronic kidney disease
ICU	Intensive care unit
INR	International normalized ratio
KDIGO	Kidney Disease: Improving Global Outcomes
NRP	Nephrotic-range proteinuria
OR	Odds ratio
