# Supplementary material for: Disease Stage-Dependent Association Between Nephrotic-Range Proteinuria and Severe Acute Kidney Injury in Patients with Liver Cirrhosis
Source: J Clin Med. 2026 May 8;15(10):3602. doi: 10.3390/jcm15103602 (PMC13207211; doi:10.3390/jcm15103602)
Supplement: Supplementary file 1 [file jcm-15-03602-s001.zip › jcm-4273604-supplementary.pdf]

Supplementary Table S1. Sensitivity and subgroup analyses for the association between nephrotic-range proteinuria and severe acute kidney injury according to sepsis status

Analysis	OR (95% CI)	P value
Overall model (original)	2.09 (1.08–4.04)	0.028
Subgroup (no sepsis)	0.92 (0.43–1.97)	0.823
Subgroup (sepsis)	0.77 (0.13–4.52)	0.768
Sensitivity (excluding sepsis)	0.92 (0.43–1.97)	0.823
Interaction (NRP)	0.93 (0.44–1.97)	0.843
Interaction (NRP × sepsis)	0.77 (0.12–4.99)	0.780

Supplementary Table S2. Multivariable logistic regression analysis for the need for renal replacement therapy incorporating hemodynamic and inflammatory variables

Variable	OR (95% CI)	P value
Age (year)	0.97 (0.90–1.03)	0.296
Male	0.14 (0.02–0.79)	0.026
DM	2.49 (0.46–13.36)	0.287
CKD	0.74 (0.07–8.26)	0.807
Albumin (g/dL)	0.37 (0.07–1.82)	0.221
Total bilirubin (mg/dL)	1.45 (0.79–2.66)	0.233
INR	3.69 (0.41–33.53)	0.246
Baseline creatinine (mg/dL)	0.38 (0.02–8.13)	0.535
Mean arterial pressure (mmHg)	1.01 (0.92–1.11)	0.778
Vasopressor use	0.36 (0.02–5.47)	0.465
Lactate (mmol/L)	3.15 (1.01–9.80)	0.047
C-reactive protein (mg/dL)	0.87 (0.63–1.20)	0.403
Child-Pugh class B vs. A	0.23 (0.03–1.95)	0.177
Child-Pugh class C vs. A	0.01 (0.00–1.66)	0.077

Odds ratios (ORs) and 95% confidence intervals (CIs) were estimated using logistic regression. All models were adjusted for age, sex, diabetes mellitus, chronic kidney disease, albumin, total bilirubin, INR, baseline creatinine, Child-Pugh class, and hemodynamic and inflammatory variables including mean arterial pressure, vasopressor use, lactate levels, and C-reactive protein.

Supplementary Table S3. Multivariable linear regression analysis for length of hospital stay incorporating hemodynamic and inflammatory variables

Variable	β (95% CI)	P value
Age (year)	-0.03 (-0.09–0.02)	0.257
Male	-1.34 (-2.88–0.21)	0.089
DM	0.88 (-0.58–2.34)	0.237
CKD	1.35 (-0.62–3.32)	0.177
Albumin (g/dL)	-0.66 (-1.78–0.47)	0.251
Total bilirubin (mg/dL)	-0.33 (-0.79–0.14)	0.169
INR	1.48 (-0.49–3.45)	0.141
Baseline creatinine (mg/dL)	0.83 (-1.80–3.46)	0.536
Mean arterial pressure (mmHg)	-0.04 (-0.12–0.05)	0.393
Vasopressor use	-1.47 (-3.94–1.00)	0.243
Lactate (mmol/L)	1.05 (-0.45–2.56)	0.168
C-reactive protein (mg/dL)	0.15 (-0.11–0.40)	0.254
Child-Pugh class B vs. A	0.47 (-1.43–2.38)	0.625
Child-Pugh class C vs. A	1.72 (-1.44–4.87)	0.286

β coefficients and 95% confidence intervals (CIs) were estimated using linear regression. All models were adjusted for age, sex, diabetes mellitus, chronic kidney disease, albumin, total bilirubin, INR, baseline creatinine, Child-Pugh class, and hemodynamic and inflammatory variables including mean arterial pressure, vasopressor use, lactate levels, and C-reactive protein.

Supplementary Table S4. Sensitivity analyses using alternative definitions of baseline creatinine

Baseline creatinine definition	Severe AKI, n (%)	Adjusted OR for NRP	95% CI	P value
Primary definition: lowest Cr	99 (24.3)	0.78	0.37–1.66	0.522
Median pre-admission Cr	101 (24.8)	0.78	0.36–1.66	0.513
Last available stable Cr	100 (24.5)	0.79	0.37–1.68	0.549

Adjusted odds ratios were estimated using multivariable logistic regression with the same covariates as in the primary analysis, including age, sex, diabetes mellitus, chronic kidney disease, albumin, total bilirubin, INR, baseline creatinine, Child–Pugh class, mean arterial pressure, vasopressor use, lactate, and C-reactive protein. NRP, nephrotic-range proteinuria; OR, odds ratio; CI, confidence interval; AKI, acute kidney injury.

Supplementary Table S5. UPCR levels and prevalence of nephrotic-range proteinuria according to AKI stage

Variable	Stage 0	Stage 1	Stage 2	Stage 3	P value
UPCR, g/g	1.75 (0.97–2.70)	1.73 (1.15–2.73)	1.75 (1.01–3.02)	1.83 (1.30–2.88)	0.862
NRP, n (%)	14 (10.1)	24 (14.1)	8 (12.3)	3 (8.8)	0.673

Values are presented as median (interquartile range) or number (percentage). Comparisons among groups were performed using the Kruskal–Wallis test for continuous variables and the chi-square test. AKI, acute kidney injury; NRP, nephrotic-range proteinuria; UPCR, urine protein-to-creatinine ratio.
